# Capturing fine-grained spatial details in brain tumor MRI via multi-module feature enhancement

**DOI:** 10.3389/fonc.2026.1875644

**Published:** 2026-08-26

**Authors:** Jianle Chen, Yuanyong Zhou, Hailin Cao, YuYan Lin, Jianyu Zhu, Huilian Liao

**Affiliations:** 1 Shunde Hospital of Guangzhou University of Chinese Medicine, Foshan, China; 2 School of Business, Macau University of Science and Technology, Macau, Macao SAR, China

**Keywords:** brain tumor, deep learning, MRI images, multi-scale features, object detection

## Abstract

Existing object detection models often face challenges in medical imaging, including limited feature fusion capabilities, constrained receptive fields, and the prevalent issue of class imbalance. To address these limitations, this paper proposes FDANet (Fine-Grained Detail Aggregation Network), a novel multi-module feature enhancement network built upon the YOLOv11 architecture. Specifically, FDANet integrates a Mixed Aggregation Network (MANet) to capture fine-grained spatial details of tumor regions. The FasterCGLU gating mechanism is employed to enhance lesion feature responses while suppressing background noise. Furthermore, a Wavelet Feature Upgrade (WFU) module is introduced to facilitate the adaptive fusion of multi-scale features. The synergy of these three components directs the model’s focus efficiently toward critical pathological regions, an efficacy substantiated by both heatmap visualizations and ablation studies. Datasets for this study were constructed by combining in-house brain tumor images with the public Alibaba Tianchi dataset. Extensive comparisons against mainstream detection models demonstrate that FDANet achieves precise identification of pituitary adenomas, gliomas, and meningiomas, attaining an mAP_50_ of 0.961. This marks a 6.0 percentage point improvement over the baseline YOLOv11, all while maintaining a low parameter count and computational complexity. This study presents a multi-module feature enhancement framework for brain tumor MRI detection, demonstrating promising performance on a single-center 2D dataset. Further prospective validation on multi-center, volumetric data is warranted before clinical translation.

## 1 Introduction

Brain tumors have a high global incidence (approximately 7–11 cases per 100,000 person-years) and are among the most common malignant tumors of the central nervous system (1). Given the unique location of their occurrence, brain tumors not only impact patient survival and prognosis but also often lead to severe impairments in personality, cognitive, and emotional functions, imposing a heavy burden on patients’ families and society as a whole (2). Epidemiological studies have shown significant disparities in the five-year survival rate of brain tumor patients: in the absence of standardized treatment, this rate is only about 15%; however, after receiving standardized comprehensive treatment, the five-year survival rate for patients can increase to nearly 90% (3). Therefore, achieving early and accurate diagnosis of brain tumors is of great significance for improving patient prognosis and reducing the disease burden.

In the field of medical image analysis, Magnetic Resonance Imaging (MRI), with its exceptional soft tissue contrast, has become an indispensable core technology for non-invasive diagnosis, clinical staging, and preoperative planning of brain tumors. With the rising incidence of brain tumors, the volume of clinically accumulated MRI data continues to grow. However, brain tumors typically exhibit significant heterogeneity, including characteristics such as irregular morphology (4), indistinct boundaries (5), and inhomogeneous signal intensity (6), posing technical bottlenecks for automatic detection and precise identification of lesions. In clinical practice, the interpretation of medical images still predominantly relies on manual assessment by radiologists. This process is not only time-consuming and labor-intensive but also highly dependent on the clinician’s level of experience, leading to significant subjectivity and inter-reader variability. The risk of misdiagnosis and missed detection is particularly high in cases involving subtle lesions or atypical presentations, which may subsequently delay critical therapeutic interventions. Consequently, traditional diagnostic approaches based primarily on visual evaluation are increasingly insufficient to meet the clinical demands for precision and efficiency in modern medicine.

The prominent advantage of deep learning technology in the medical field lies in its ability to automatically learn and extract high-level, deep-seated features from massive amounts of raw medical data (7), thereby eliminating the reliance on manually designed features and prior knowledge (8). Leveraging its powerful high-dimensional data processing capabilities, deep learning can uncover nonlinear correlations and lesion heterogeneity information that is difficult to discern with the human eye. Consequently, it achieves accuracy approaching or even surpassing that of human experts in tasks such as lesion segmentation (9) and prognosis prediction (10). Furthermore, this technology can significantly enhance the automation level and efficiency of the diagnostic process. By rapidly processing large-scale imaging data, it assists physicians in early screening and risk stratification, reducing misdiagnoses and missed detections caused by variations in subjective experience. In summary, deep learning is driving the transformation of medical diagnosis from a traditional experience-based model to a data-driven, intelligent paradigm.

## 2 Related work

For medical image analysis, object detection algorithms with Convolutional Neural Networks (CNNs) as their core have demonstrated significant advantages in the automatic identification and precise localization of brain tumors. Deep network architectures such as U-Net (11) have achieved detection accuracy exceeding 90% on public datasets (12). These algorithms adopt an end-to-end learning paradigm, enabling them to automatically extract multi-scale features from images, which allows for the effective capture and identification of subtle structural differences in tumor tissues and significantly enhances the perceptual capability regarding heterogeneous lesion manifestations.

In the task of brain tumor detection, researchers continuously refine and innovate new techniques. Ming Kang et al. (13) integrated a bi-level routing attention mechanism, a generative feature pyramid network, and a fourth detection head into YOLOv8, achieving promising results on the Br35H dataset. However, this work was limited to public datasets and was not validated in actual hospital settings, leaving its generalizability open to question. Chengkun Hong et al. (14) constructed a suitable brain tumor dataset and performed brain tumor medical image segmentation and detection based on the YOLOv11 model. In 2024, Wang Z. et al. (15) further proposed the Mamba-YOLO model, which introduces a State Space Model (SSM) to effectively alleviate the computational pressure caused by the quadratic complexity of the self-attention mechanism. Mamba-YOLO optimizes the basic SSM structure and adapts it for object detection tasks by designing LSBlock and RGBlock modules, enhancing the ability to capture local dependencies in images and significantly improving the model’s robustness and detection performance. Shah et al. (16) proposed a fine-tuned model based on EfficientNet-B0, which coordinates the network’s depth, width, and resolution through a compound scaling coefficient, significantly compressing the model size while maintaining high detection accuracy, thereby offering a feasible solution for deploying medical image analysis on edge devices. In 2023, Wang C. et al. (17) introduced the Gold-YOLO object detection model, whose core innovation lies in the Aggregation-Distribution (GD) mechanism. By fusing multi-layer features and injecting global information into high-level representations, it achieves efficient information interaction and transmission within the YOLO architecture. Ma Yangyuan et al. (18) proposed a brain tumor anomaly detection algorithm based on an improved diffusion model, incorporating a ConvNeXt Block structure and a fast ordinary differential equation solver to effectively model the distribution of healthy data and enhance the detection efficiency of anomalous samples.

In contrast, S. Lincy Jemina et al. (19) proposed a hybrid lightweight deep learning framework (HybLwDL) that integrates a twin-attentive pyramid convolutional network (LHTA-PCNet) with Stellar Oscillation Optimizer (SOO) and Grad-CAM visualization, achieving 99.5% accuracy on the BT Detection 2020 dataset. In a separate study, the same authors (20) proposed an improved optimization-based model (ImpOML) using an Extended Sea-Horse assisted MLP (ExSH-MLP) optimized by an enhanced sea-horse optimization (ESHO) algorithm, achieving 98.5% accuracy on the BraTS-2021-task1 dataset. For brain tumor segmentation, Surendran Rajendran et al. (21) proposed a combinatorial strategy merging two networks (U-Net and 3D CNN) combined with GLCM-based feature extraction. Their method achieved high precision (99.41%), F-score (99.40%), and sensitivity (99.39%) for whole tumor segmentation on the validation set.

While the aforementioned deep learning-based methods for brain tumor detection have, to some extent, alleviated many issues in traditional diagnostic workflows and achieved significant progress in detection accuracy and computational efficiency, they still face several limitations that need to be addressed in practical clinical applications. These are primarily manifested in the following aspects: (1) Limited receptive field expansion. The receptive field expansion capability of existing models is relatively insufficient. For diffuse tumors with blurred boundaries and scattered distributions, it is challenging for these models to fully capture the global structural characteristics and key contextual information of the lesions, often leading to incomplete detection results or deviations in lesion boundary localization. (2) Insufficient feature fusion capabilities. When processing complex backgrounds and multi-scale targets, the feature fusion capabilities of current models still need further enhancement to better handle the high variability of brain tumors in terms of morphology, size, and location. (3) Data class imbalance. Current research primarily evaluates models based on public datasets, which commonly exhibit imbalanced class distributions. This limits the models’ generalization ability and robustness in real-world clinical scenarios. To bridge this gap between research benchmarks and clinical practice, the present study incorporates institutional clinical data alongside public datasets.

To address the aforementioned limitations, this paper proposes corresponding improvement strategies from three dimensions: data augmentation, network structure optimization, and feature fusion. The contributions of this article are as follows: (1) To mitigate the issues of imbalanced class distribution and insufficient sample diversity in public datasets, this paper integrates public datasets with clinical imaging data from our hospital during the model training phase. A hybrid augmentation strategy is employed to enrich the distribution characteristics of samples, thereby enhancing the model’s generalization ability and robustness in real-world clinical scenarios. (2) To tackle the problem of limited receptive field expansion and the difficulty in perceiving multi-scale targets, this paper introduces a Mixed Aggregation Network (MANet) (22). By embedding an attention mechanism into the feature extraction process, the model’s ability to model the context of diffuse tumors’ global structure and blurred boundary regions is strengthened, thereby improving the accuracy of lesion integrity detection and boundary localization. (3) To enhance the model’s feature representation capability in complex backgrounds and sensitivity to subtle structural differences, this paper adopts the FasterCGLU activation function to replace traditional activation units, leveraging its superior non-linear mapping capabilities to accelerate network convergence. (4) To address the issue of insufficient multi-scale feature fusion, this paper introduces a Wavelet Feature Upgrade (WFU) module (23). By adaptively weighting and fusing features from different levels, the model’s representation capability for the high variability in brain tumor morphology, size, and location is optimized. It is worth noting that the idea of wavelet domain feature enhancement in this study (24) is similar to the concept of the WFU module in this paper. However, the two solve different problems, pointing out that they use wavelet transform to suppress noise and enhance tumor boundaries.

Beyond the simple aggregation of existing modules, the core novelty of FDANet lies in the principled synergy of three complementary feature enhancement mechanisms. Unlike typical YOLO-based detectors that rely on deeper networks or larger kernels to expand receptive fields, our MANet extracts multi-scale spatial details through parallel paths without increasing depth. The FasterCGLU gating mechanism is distinct from standard activation functions (ReLU, SiLU) in that it learns to selectively enhance lesion-relevant features while actively suppressing background noise. Furthermore, the WFU module performs wavelet-domain cross-scale fusion, which is rarely adopted in mainstream detection frameworks. To our knowledge, no prior work has combined these three strategies (multi-path parallel extraction, gated feature screening, and wavelet-domain fusion) specifically for brain tumor MRI detection. This unique combination, together with its low computational overhead, constitutes the primary methodological contribution of this study.

## 3 Methodology

**Figure 1:**
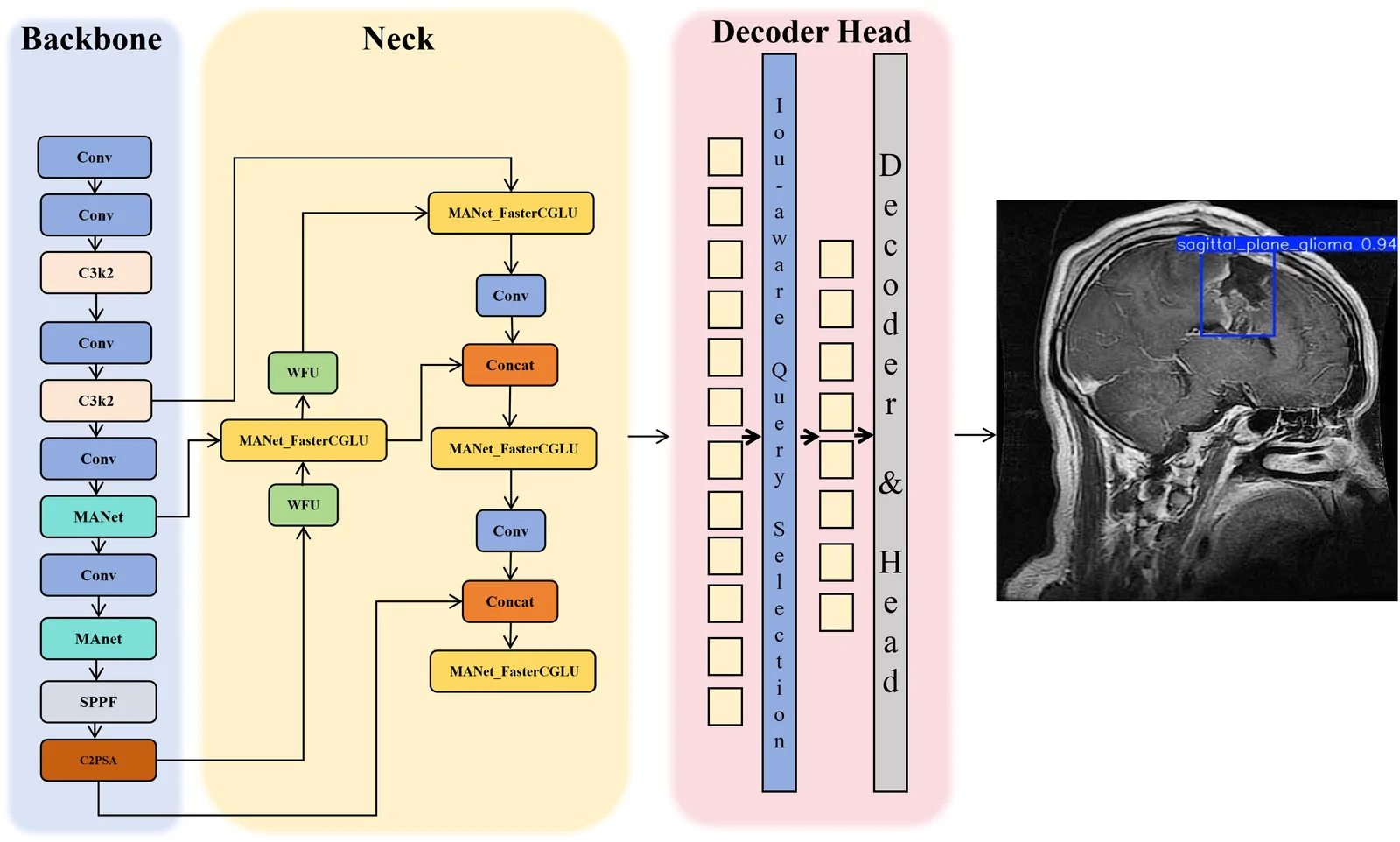
The overall architecture of FDANet.

Brain tumor MRI detection faces several challenges. These challenges include difficulty in identifying small lesions, low precision in delineating irregular boundaries, and significant interference from background noise. To address these issues, this paper proposes a multi-module collaborative enhancement network. This network is named FDANet (**F**ine-Grained **D**etail Aggregation **N**etwork). Before detailing each module, this paper briefly summarizes how the three proposed components work together. As illustrated in **Figure 1**, FDANet builds upon YOLOv11. In the backbone, the MANet module replaces the original C3k2 to capture multi-scale spatial details. In the neck, the WFU module first adaptively fuses shallow detail features and deep semantic features. Then, a MANet_FasterCGLU module further processes the fused features. Its gating mechanism enhances tumor-relevant responses while suppressing background noise. The three modules operate in a pipeline without nested loops, and each module’s output serves directly as the next module’s input.

### 3.1 Wavelet feature upgrade module

In brain tumor image recognition tasks, input images are typically derived from Computed Tomography (CT) or Magnetic Resonance Imaging (MRI). These images are characterized by a homogeneous background (uniform black or grayscale) and clearly defined objects of interest (tumor regions with relatively distinct boundaries but variable scales). Assume the two-level feature maps extracted by the backbone network are defined as follows: the high-resolution detail feature $F_{1}\in {\mathbb{R}}^{H\times W\times C}$ (where *H* and *W* are the spatial dimensions, and *C* is the number of channels) and the low-resolution semantic feature $F_{2}\in {\mathbb{R}}^{\frac{H}{2}\times \frac{W}{2}\times 2C}$. Due to translation invariance, traditional convolutional layers compute responses densely across the entire image. As a result, *F*_1_ contains a substantial amount of redundant or irrelevant background features (such as uniform background noise and repetitive organ textures). This redundant information occupies the capacity of the feature map without contributing beneficially to tumor detection. When these redundant features are fed into subsequent fusion networks (such as PANet or BiFPN), the fusion operation is delayed—the network must first suppress irrelevant features before it can focus on tumor regions. This leads to insufficient multi-scale fusion: shallow details fail to interact with deep semantics in a timely manner, ultimately resulting in missed detection of small tumors, blurred boundaries, and low classification confidence.

**Figure 2:**
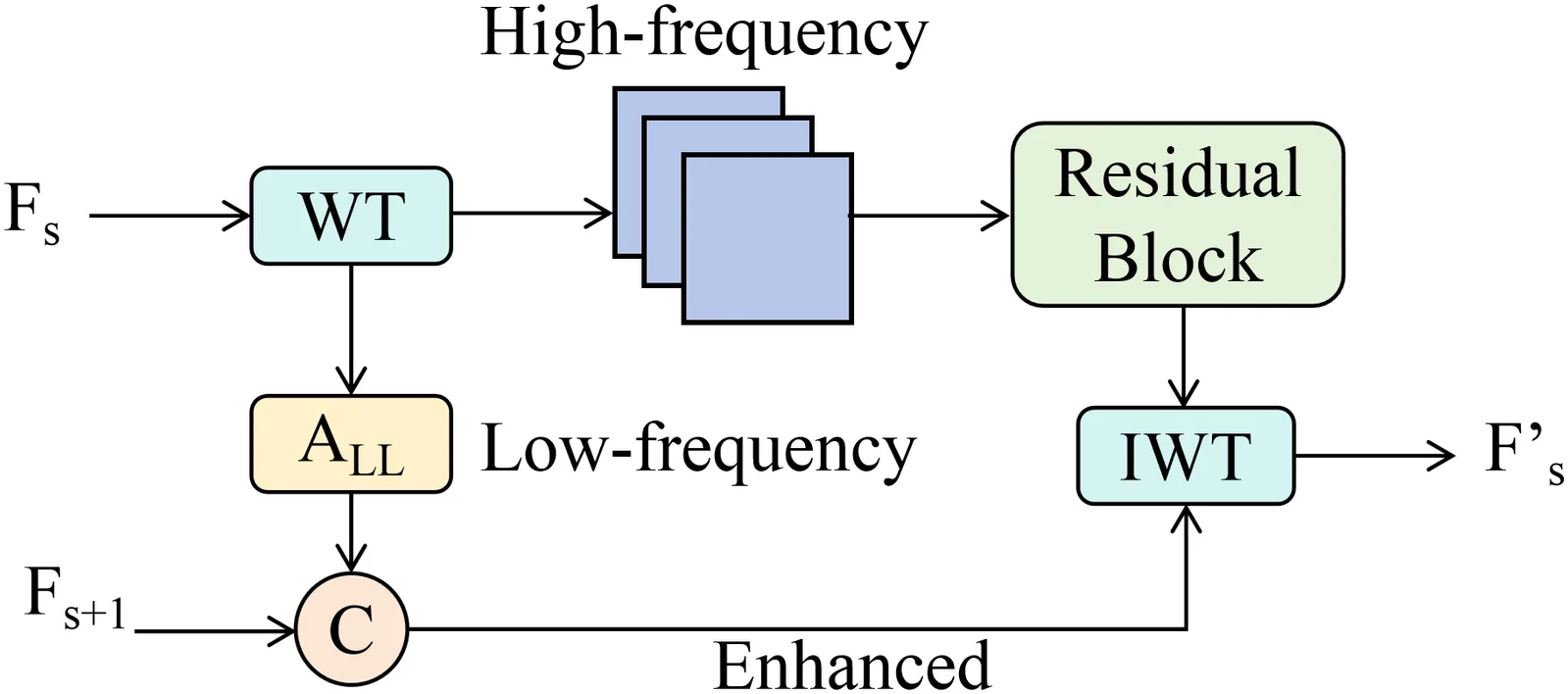
The architecture of the Wavelet Feature Upgrade (WFU) module.

To address this issue, this paper introduces WFU (Wavelet Feature Upgrade), with its schematic diagram shown in **Figure 2**. First, a discrete wavelet transform WT(·) is applied to the high-resolution feature *F*_1_, decomposing it into one low-frequency approximation component and three high-frequency detail components, as shown in Equation 1:

(1) $$\left\{A_{LL},H_{LR},V_{RL},D_{RR}\right\}=WT\left(F_{1}\right)$$

In the equation, $A_{LL}\in {\mathbb{R}}^{\frac{H}{2}\times \frac{W}{2}\times C}$ represents the low-frequency component, corresponding to the main structure of the tumor. $H_{LR},V_{RL},D_{RR}\in {\mathbb{R}}^{\frac{H}{2}\times \frac{W}{2}\times C}$ are the high-frequency detail components in the horizontal, vertical, and diagonal directions, respectively, containing edge and noise information.

As shown in Equation 2, to suppress background redundancy in the high-frequency components, the three high-frequency components are summed to obtain a comprehensive high-frequency feature.

(2) $$H_{sum}=H_{LR}+V_{RL}+D_{RR}\in {\mathbb{R}}^{\frac{H}{2}\times \frac{W}{2}\times C}$$

Next, a learnable redundancy suppression gate *G* is generated through a 1×1 convolutional layer and the Sigmoid activation function σ(·), as shown in Equation 3:

(3) $$G=\sigma \left(Conv\left(H_{sum}\right)\right)\in {\mathbb{R}}^{\frac{H}{2}\times \frac{W}{2}\times C}$$

Here, Conv(·) denotes the convolution operation. Each element of the gate signal *G* takes a value in the range [0,1], representing the retention weight of the high-frequency feature at the corresponding position.

The gate *G* is multiplied element-wise with the comprehensive high-frequency feature to obtain the enhanced high-frequency feature *H*′, as shown in Equation 4:

(4) $$H^{\prime }=G⊙H_{sum}\in {\mathbb{R}}^{\frac{H}{2}\times \frac{W}{2}\times C}$$

In the equation, ⊙ denotes the Hadamard product.

To achieve low-frequency-dominated cross-scale fusion, the low-resolution semantic feature *F*_2_ is first upsampled to match the spatial dimensions of the low-frequency component, resulting in the upsampled semantic feature $F_{2}^{↑}$, as shown in Equation 5:

(5) $$F_{2}^{↑}=Up\left(F_{2}\right)\in {\mathbb{R}}^{\frac{H}{2}\times \frac{W}{2}\times 2C}$$

Here, *Up*(·) represents bilinear interpolation upsampling. The low-frequency component *A_LL_* is then concatenated with the upsampled semantic feature $F_{2}^{↑}$ along the channel dimension, as shown in Equation 6:

(6) $$F_{fuse}=∁\left(A_{LL},F_{2}^{↑}\right)\in {\mathbb{R}}^{\frac{H}{2}\times \frac{W}{2}\times 3C}$$

Here, ∁(·,·) denotes the concatenation operation.

Finally, as shown in Equation 7, the fused low-frequency feature *F_fuse_* and the enhanced high-frequency feature *H*′ are jointly fed into the inverse discrete wavelet transform $WT^{-1}\left(\cdot \right)$ to reconstruct the high-resolution output feature *F_out_*:

(7) $$F_{out}=WT^{-1}\left(F_{fuse},H^{\prime }\right)\in {\mathbb{R}}^{H\times W\times C}$$

Through the aforementioned formalized process, the WFU module obtains clean and information-rich multi-scale features at an early stage. This effectively enhances the detection performance of YOLOv11 on brain tumor images. The detailed computation flow of the WFU module is presented in **Algorithm 1**.

Algorithm 1. Forward pass of Wavelet Feature Upgrade (WFU) module 
Input: High-resolution feature F1 ∈ ℝ^{H×W×C}^,
      Low-resolution semantic feature F2 ∈ ℝ^{H/2×W/2×2C}^
Output: Enhanced feature Fout ∈ ℝ{H×W×C}
1:   ▷ Discrete wavelet transform
2:   A_LL, H_LR, V_RL, D_RR ← DWT(F1)    ▷ Equation 1
3:
4:   ▷ High-frequency aggregation
5:   H_sum ← H_LR + V_RL + D_RR       ▷ Equation 2
6:
7:   ▷ Learnable redundancy suppression gate
8:   G ← Sigmoid(Conv1×1(H_sum))     ▷ Equation 3
9:   H’ ← G ⊙ H_sum           ▷ Equation 4, element-wise product
10:
11: ▷ Low-frequency cross-scale fusion
12: F2_up ← BilinearUpsample(F2, size=H/2)  ▷ Equation 5
13: F_fuse ← Concat(A_LL, F2_up, dim=C)   ▷ Equation 6
14:
15: ▷ Inverse wavelet transform for reconstruction
16: F_out_ ← IDWT(F_fuse_, H’)           ▷ Equation 7
17: return F_out_

### 3.2 MANet mixed aggregation modules

**Figure 3:**
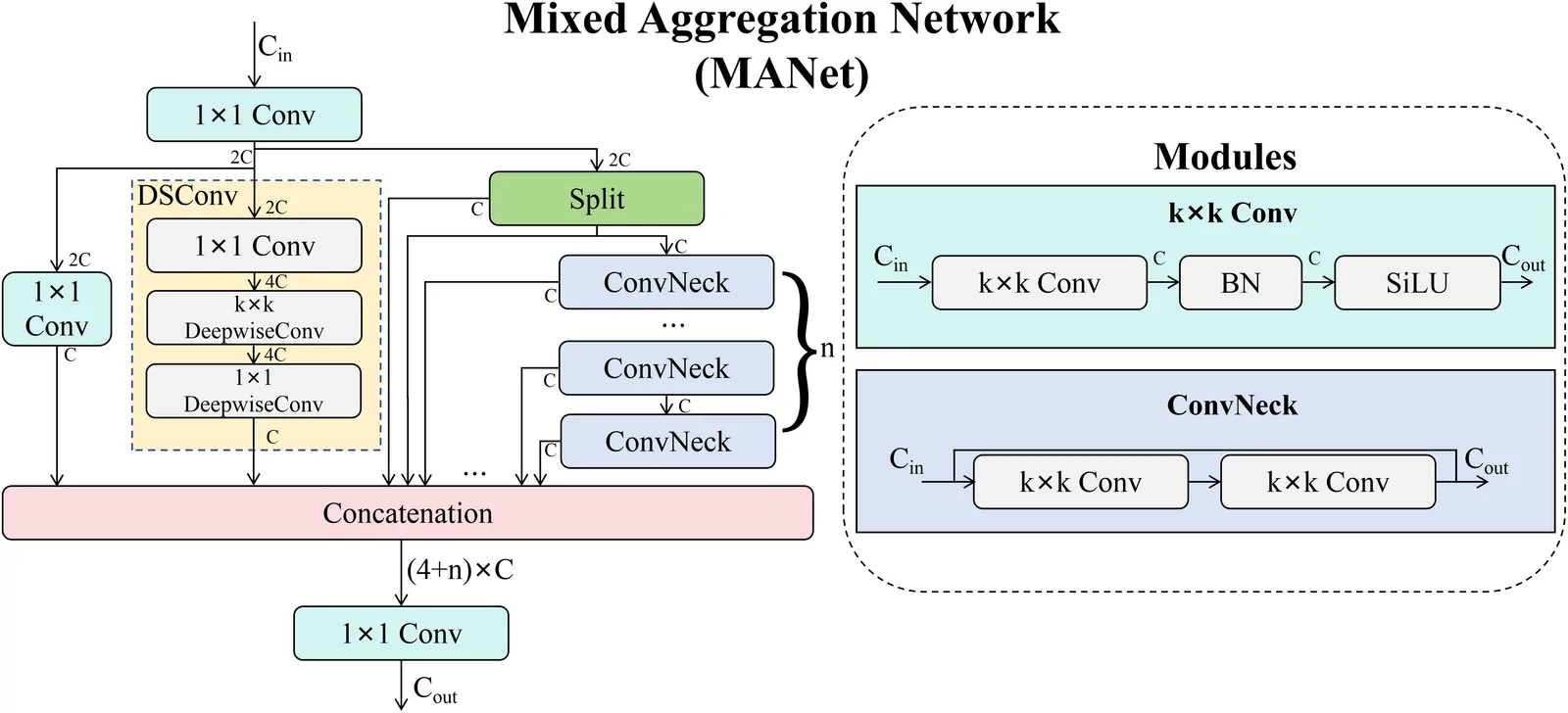
Illustration of the proposed mixed aggregation network (MANet).

To enhance the model’s ability to perceive small lesions and irregular boundaries in brain tumor detection, this paper introduces the Mixed Aggregation Network (MANet) into the backbone network. The core design philosophy of MANet lies in adaptively capturing critical spatial details by fusing feature paths with different receptive fields in parallel, as illustrated in **Figure 3**. Its specific operations can be described by Equations 9 and 10.

First, the input feature map *X_in_* passes through a 1×1 convolution to generate an intermediate feature X_mid_ (with the number of channels expanded to 2*C*), laying the foundation for subsequent multi-path processing, as shown in Equation 8:

(8) $$X_{\mathrm{mid}}=Conv_{1}\left(X_{in}\right)$$

Subsequently, MANet constructs three feature extraction paths in parallel, each with different receptive fields: Path 1 employs a regular 3×3 convolution to capture global contextual information: $X_{1}=Conv_{2}\left(X_{\mathrm{mid}}\right)$; Path 2 strengthens the modeling of local spatial details through depthwise separable convolution (DSConv): $X_{2}=\text{DSConv}({\text{Conv}}_{3}(X_{mid}))$; Path 3 splits *X_mid_* into *X_3_* and *X_4_*, and applies a series of residual-connected ConvNeck blocks to X_4_, progressively refining the feature representation. The expression for ConvNeck is given in Equation 9. Here, *X_3_* directly participates in the final concatenation as the original feature copy retained after the Split operation. It bypasses the ConvNeck chain and is fed directly into the concatenation layer, forming a multi-scale, multi-level feature set together with the outputs from the other paths. Finally, a 1×1 convolution compresses the concatenated features to produce the output *X_out_* of MANet. The mathematical expression for MANet is provided in Equations 10 and 11:

(9) ConvNeck(x)=Conv(BN(ReLU(x)))+x

(10) $$\{\begin{array}{c} X_{5}=ConvNeck_{1}\left(X_{4}\right)+X_{4}\\ X_{6}=ConvNeck_{2}\left(X_{5}\right)+X_{5}\\ \cdots ,\\ X_{4+n}=ConvNeck_{n}\left(X_{3+n}\right)+X_{3+n} \end{array}$$

Through multiple residual stacking operations, this process effectively preserves shallow details while enriching deep semantics, which is particularly beneficial for delineating the blurred boundaries of brain tumors.

The three aforementioned paths focus on information at different scales: the standard convolution provides stable semantic primitives, the depthwise separable convolution is highly sensitive to textural variations in small lesions, and the residual chain adaptively integrates multi-level features. In the context of brain tumor detection, tumor sizes can range from metastatic foci of a few millimeters to space-occupying lesions of several centimeters, and their morphology is often highly irregular. Through its parallel processing design, MANet enables the network to dynamically adjust the contribution weights of each path based on the input image content (such as tumor size and morphology), thereby achieving adaptive capture of critical spatial details.

Finally, the output feature maps from all paths are concatenated along the channel dimension and compressed by a 1×1 convolution to obtain the final enhanced feature X*_out_*, as shown in Equation 11:

(11) $$X_{out}=Conv_{0}\left(X_{1}∥X_{2}∥\cdots ∥X_{4+n}\right)$$

The fusion operation of this module not only aggregates multi-scale information but also further highlights the response to critical brain tumor regions through learnable weight allocation. Equation 10 is responsible for generating diverse intermediate features, providing rich semantic and detailed information for subsequent fusion. Equation 11, in turn, effectively integrates these features, adaptively emphasizing region responses critical to the target task through learnable convolution weights. Together, they embody the complete design philosophy of the MANet module, spanning from “multi-path extraction” to “adaptive fusion”. The detailed computation flow of the WFU module is presented in **Algorithm 2**.

Algorithm 2. Forward Pass of MANet module 
Input: Feature map Xin ∈ ℝ^{H×W×C1}^
Output: Enhanced feature map Xout ∈ ℝ^{H×W×C2}^
Parameters: expansion ratio e, number of Bottleneck blocks n
1:   c ← C2 × e         ▷ Intermediate channels
2:   *X_mid* ← Conv1×1(X_in, 2C)    ▷ Equation 8, X_mid
3:
4:   ▷ Path 1: 1×1 convolution branch
5:   X0 ← Conv1×1(X, c)          ▷ cv_block_1
6:
7:   ▷ Path 2: Depthwise separable convolution branch
8:   X_temp ← Conv1×1(X, dim_hid)   ▷ cv_block_2 part 1
9: X_temp ← DWConv3×3(X_temp)    ▷ cv_block_2 part 2
10:   X1 ← Conv1×1(X_temp, c)    ▷ cv_block_2 part 3
11:
12:   ▷ Path 3: Split and residual chain branch
13:   X2, X3 ← Split(X, dim=1)   ▷ Two halves: each of size c
14:   X ← Concatenate(X0, X1, X2, X3)  ▷ List initialization
15:
16:   ▷ Apply n Bottleneck blocks in sequence
17:   for i ← 1 to n do
18:   X3 ← Bottleneck(X3)    ▷ onvNeck with residual
19:     X ← Append(X3)
20:   end for
21:
22:   ▷ Final fusion
23:   X_out ← Conv1×1(Concatenate(X), C2)    ▷ Equation 11
24:   return X_out

### 3.3 MANet-FasterCGLU

Brain tumors on MRI often exhibit characteristics such as low grayscale contrast and blurred boundaries. Traditional activation functions struggle to effectively enhance these subtle features in shallow networks, leading to insufficient response in lesion regions. Additionally, small lesions are prone to being lost in deep networks, while large lesions rely on global contextual information for accurate localization. Therefore, based on the MANet architecture described in Section 3.2, this paper replaces the ConvNeck residual chain in Path 3 with a FasterCGLU block to form the MANet-FasterCGLU module. The multi-path extraction (Paths 1 and 2) remains unchanged. The FasterCGLU block introduces a learnable gating mechanism, whose expression is given in Equation 12:

(12) $$y=x\times \sigma \left({\text{W}}_{1}x+{\text{b}}_{1}\right)+\left(1-\sigma \left({\text{W}}_{1}x+{\text{b}}_{1}\right)\right)\times tanh\left({\text{W}}_{2}x+{\text{b}}_{2}\right)$$

Here, *x* denotes input feature vector (or the channel vector at a specific position in a feature map). In the MANet-FasterCGLU module, *x* is the part of the feature that is sent to the gating unit after splitting the path. *y* denotes output feature, with the same shape as *x*. It is the result of dynamically weighted fusion between the original input *x* and the nonlinearly transformed features. ⊙denotes element-wise multiplication (hadamard product), i.e., multiplying corresponding elements. W_1_*x*, b_1_ denotes learnable weight matrix and bias vector that act on *x* to produce the gating signal. σ(·) denotes Sigmoid activation function, with output range (0,1), used to generate the gating weights. tanh(·) denotes hyperbolic tangent activation function, with output range (-1,1), used to normalize the transformed features. W_2_*x*, b_2_ denotes Another set of learnable weights and biases, used to linearly transform *x* before feeding it into tanh.

**Figure 4:**
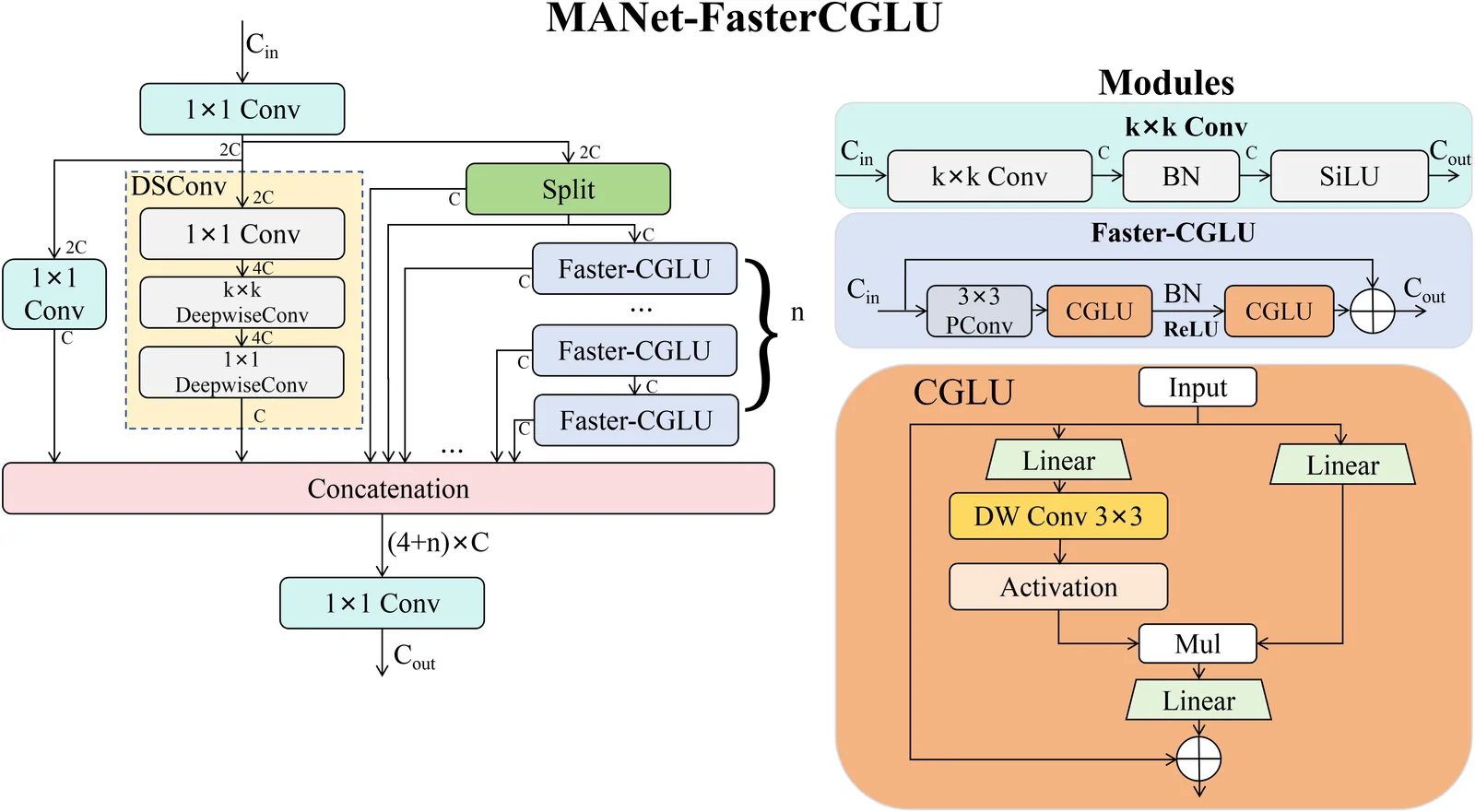
MANet-FasterCGLU.

This gating adaptively enhances tumor-region features while suppressing background noise. **Algorithm 3** details the forward pass. The overall structure is shown in **Figure 4**.

Algorithm 3. Forward pass of MANet-FasterCGLU module 
Input: Feature map x ∈ℝ^{B×C×H×W}
Output: Gated feature map y ∈ ℝ^{B×C×H×W}
Parameters: convolution kernels W1, W2; biases b1, b2
1:   ▷ Step 1: Compute gate weights (sigmoid branch)
2:   s ← Conv1×1 (x, W1) + b1  ▷ Linear transform
3:   g ← σ(s)  ▷ Sigmoid activation, Equation 12 first term
4:
5:   ▷ Step 2: Compute transformed features (tanh branch)
6:   t ← Conv1×1(x, W2) + b2    ▷ Linear transform
7:   h ← tanh(t)  ▷ Tanh activation, Equation 12 second term
8:
9:   ▷ Step 3: Adaptive gated fusion
10:    y ← g ⊙ x + (1 - g) ⊙ h   ▷ Equation 12: weighted combination
11:
12:   return y

## 4 Experiments

This study integrates public datasets with clinical imaging data from our hospital to conduct a comprehensive comparison between the proposed detection method and existing algorithms, including visual comparisons of detection results. Subsequently, the performance contribution of each core module of the model is validated through systematic ablation experiments. Finally, real medical images retrieved from the hospital database are used for testing.

### 4.1 Dataset and preprocessing

The dataset in this study consists of two parts: a public dataset and an in-house dataset. The public dataset is sourced from the Aliyun Tianchi Public Dataset (25), from which 1,104 images were selected. As this dataset is open-source and designed to protect patient privacy, the original data do not provide patient-level details.

The in-house dataset was obtained from the PACS Imaging Center of Shunde Hospital, Guangzhou University of Chinese Medicine, with data collected between 2018 and 2026. All in-house images were acquired using T1-weighted contrast-enhanced sequences. Two MRI scanners were used: a Philips ACHIEVA 1.5T (Netherlands) and a Siemens MAGNETOM VIDA 3.0T (Germany). Slice spacing parameters varied by tumor type and imaging plane. For meningioma and glioma, the transverse plane was acquired at 6 mm intervals, the sagittal plane at 2 mm intervals, and the coronal plane at 6 mm intervals. For pituitary tumor, the sagittal and coronal planes were acquired at 2 mm intervals each, while the transverse plane was not uniformly acquired. Specifically, from 2022 to 2026, a total of 141 patients were enrolled. From 2018 to 2021, a total of 211 patients were enrolled. The overall in-house cohort comprised 352 patients (189 male, 163 female; age range 18–82 years, mean 54.3 ± 15.2). For each patient, scans were acquired in the transverse, sagittal, and coronal planes, with one representative image taken from each plane. Due to clinical cost considerations and the need to reduce scanning time, some patients underwent scanning in only two of the three planes.

**Figure 5:**
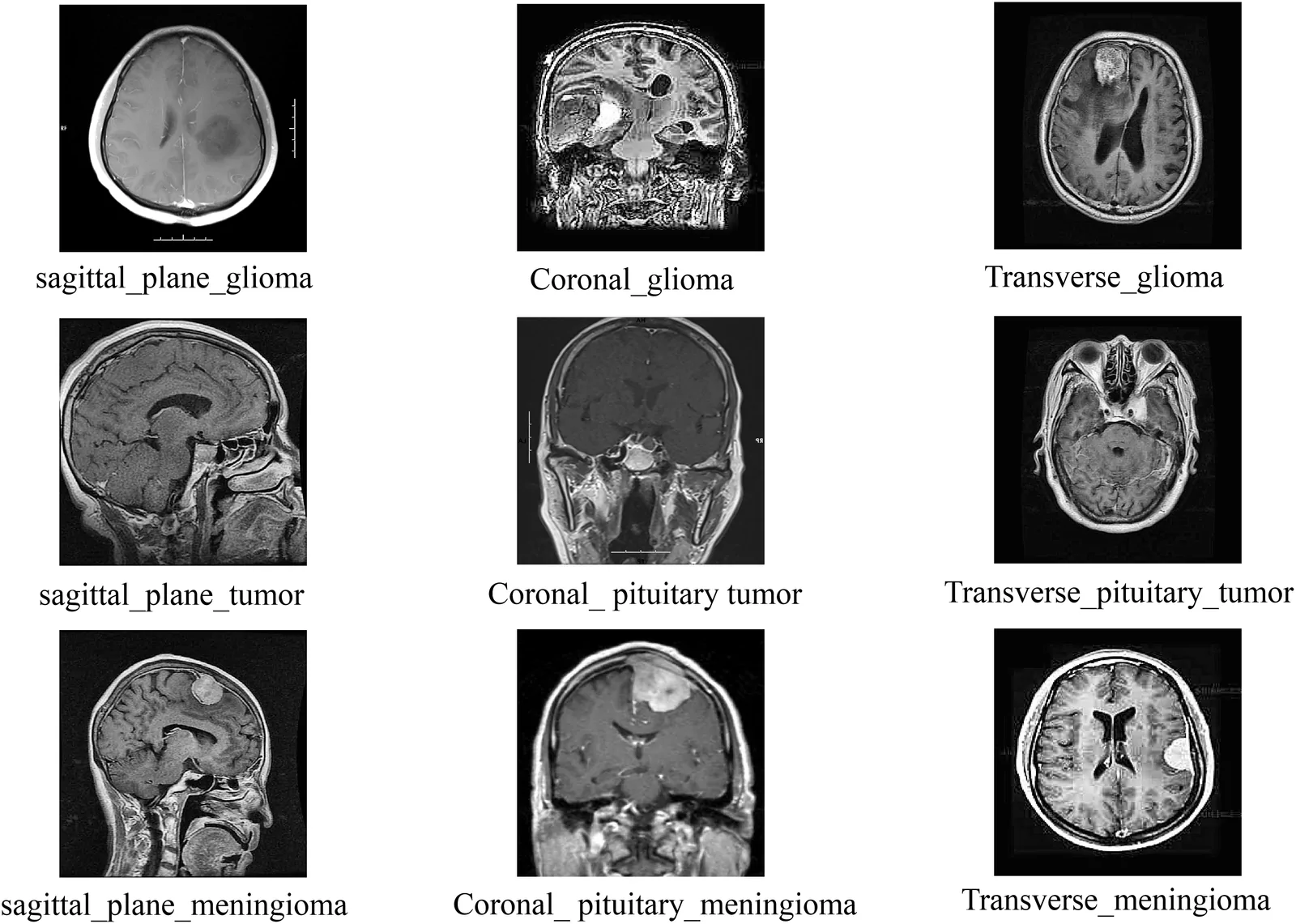
Dataset composition. The dataset contains nine categories: sagittal glioma, coronal glioma, transverse glioma, sagittal pituitary tumor, coronal pituitary tumor, transverse pituitary tumor, sagittal meningioma, coronal meningioma, and transverse meningioma.

All images including the public dataset and the in-house dataset were annotated by a senior physician from the imaging center (the second author, holding the title of Associate Chief Physician) based on imaging characteristics, resulting in a total of nine categories, as shown in **Figure 5**. These categories are: sagittal_plane_glioma, Coronal_glioma, Transverse_glioma, sagittal_plane_tumor, Coronal_pituitary_tumor, Transverse_pituitary_tumor, sagittal_plane_meningioma, Coronal_meningioma, and Transverse_meningioma.

Regarding dataset partitioning, this study strictly adopted a patient-level strategy to ensure that all images from the same patient appear in only one subset—either the training set, validation set, or test set—thereby avoiding data leakage. Specifically, after merging the Aliyun Tianchi public dataset with the in-house data from 2022-2026, the combined data were randomly partitioned into a training set (920 images), a validation set (263 images), and a test set (132 images). To enhance the robustness of model training, data augmentation was applied only to the training set after partitioning, using methods such as random rotation and symmetric flipping. After augmentation, the training set increased to 3,156 images, while the validation set and test set remained at 263 and 132 images, respectively, to ensure unbiased evaluation of model generalization. The specific number of datasets is shown in **Table 1**.

**Table 1:** The total sample count.

Class name	Train	Val	Test	Total
sagittal_plane_glioma	124	36	16	176
Coronal_glioma	112	39	11	162
Transverse_glioma	211	54	14	279
sagittal_plane_tumor	89	21	9	119
Coronal_ pituitary tumor	83	22	17	122
Transverse_pituitary_tumor	91	18	14	123
sagittal_plane_meningioma	47	20	6	73
Coronal_ pituitarymeningioma	60	20	17	97
Transverse_meningioma	103	33	28	164
Total	920	263	132	1315

In addition, the 211 in-house patients collected between 2018 and 2021 were used as an independent generalization test set to evaluate the model’s generalization ability.

### 4.2 Experimental setting and evaluation metrics

#### 4.2.1 Experimental settings

As shown in **Table 2**, the input resolution was set to 640×640, and the SGD optimizer with an initial learning rate of 0.01 was used. All models were trained with the same hyperparameters.

**Table 2:** Core hyperparameters used in the experiments.

Category	Parameter	Value/description
Software & Hardware	GPU	NVIDIA GeForce RTX 5060 Ti
	CUDA/PyTorch	CUDA 12.8, PyTorch 2.7.1, Python 3.11
Input & Training	Input resolution	640×640
	Batch size	16
	Epochs	300
	Optimizer	SGD
	Initial learning rate	0.01
	Learning rate decay factor	0.01
	Momentum	0.937
	Weight decay	0.0005
	Warm-up epochs	3

#### 4.2.2 Evaluation metrics

Several commonly used evaluation metrics in the fields of medical imaging and object detection were employed to assess the performance of the proposed object detection method. These metrics include Precision (P), Recall (R), mean Average Precision (mAP), and the number of model parameters. The formulas for “Precision” and “Recall” are provided in Equations 13 and 14:

(13) $$P=\frac{TP}{TP+FP}$$

(14) $$R=\frac{TP}{TP+FN}$$

*TP* is the positive sample correctly detected by the model. *FP* is the positive sample detected by model error detection. *FN* stands for False Negative.

AP (Average Precision) is an indicator used to measure the detection accuracy of the model. It reflects the average performance accuracy across different categories by calculating the area under the Precision-Recall (P-R) curve. The calculation formula for Average Precision (AP) is shown in 15:

(15) $$AP=\int_{0}^{1}pre\left(rec\right)drec$$

Here, *pre* is Precision, which refers to the proportion of samples predicted as positive by the model that are truly positive. *rec* is Recall, which refers to the proportion of all true positive samples correctly predicted by the model.

The mAP (mean Average Precision) is the average of all categories of AP, obtained by summing and averaging each AP value. The calculation formula is shown in Equation 16:

(16) $$mAP=\frac{sum\left(AP\right)}{n}$$

To further analyze the characteristics of the model, this study evaluates it from two dimensions: computational complexity and structural efficiency. The parameter count (Parameters) reflects the model capacity, while the number of Giga Floating-Point Operations (GFLOPs) represents the computational cost. The formulas for calculating model parameters and GFLOPs are provided in Equations 17 and 18, respectively:

(17) Params=[i×(k×k)×o]+o

(18) GFLOPs=2×H×W×Params

### 4.3 Experimental results

#### 4.3.1 Tumor detection experiments

To validate the effectiveness of the FDANet model in brain tumor detection tasks, this study comprehensively evaluated model performance from four dimensions: Precision, Recall, mean Average Precision (mAP_50_), and the comprehensive metric across IoU thresholds (mAP_50-95_). Experiments were conducted on a medical imaging dataset containing multi-planar and multi-tumor types, with detailed results presented in **Table 3**.

**Table 3:** Detection results for different categories of brain tumors.

Class name	Precision	Recall	mAP_50_	mAP_50-95_
Average	0.96	0.952	0.961	0.874
sagittal_plane_glioma	1	0.969	0.995	0.93
Coronal_glioma	0.896	0.929	0.897	0.84
Transverse_glioma	0.918	0.916	0.975	0.846
sagittal_plane_tumor	0.987	1	0.995	0.905
Coronal_pituitary_tumor	0.988	1	0.995	0.858
Transverse_pituitary_tumor	0.908	0.991	0.98	0.807
sagittal_plane_meningioma	0.976	0.909	0.925	0.879
Coronal_pituitarymeningioma	0.983	0.905	0.934	0.899
Transverse_meningioma	0.979	0.948	0.955	0.903

**Figure 6:**
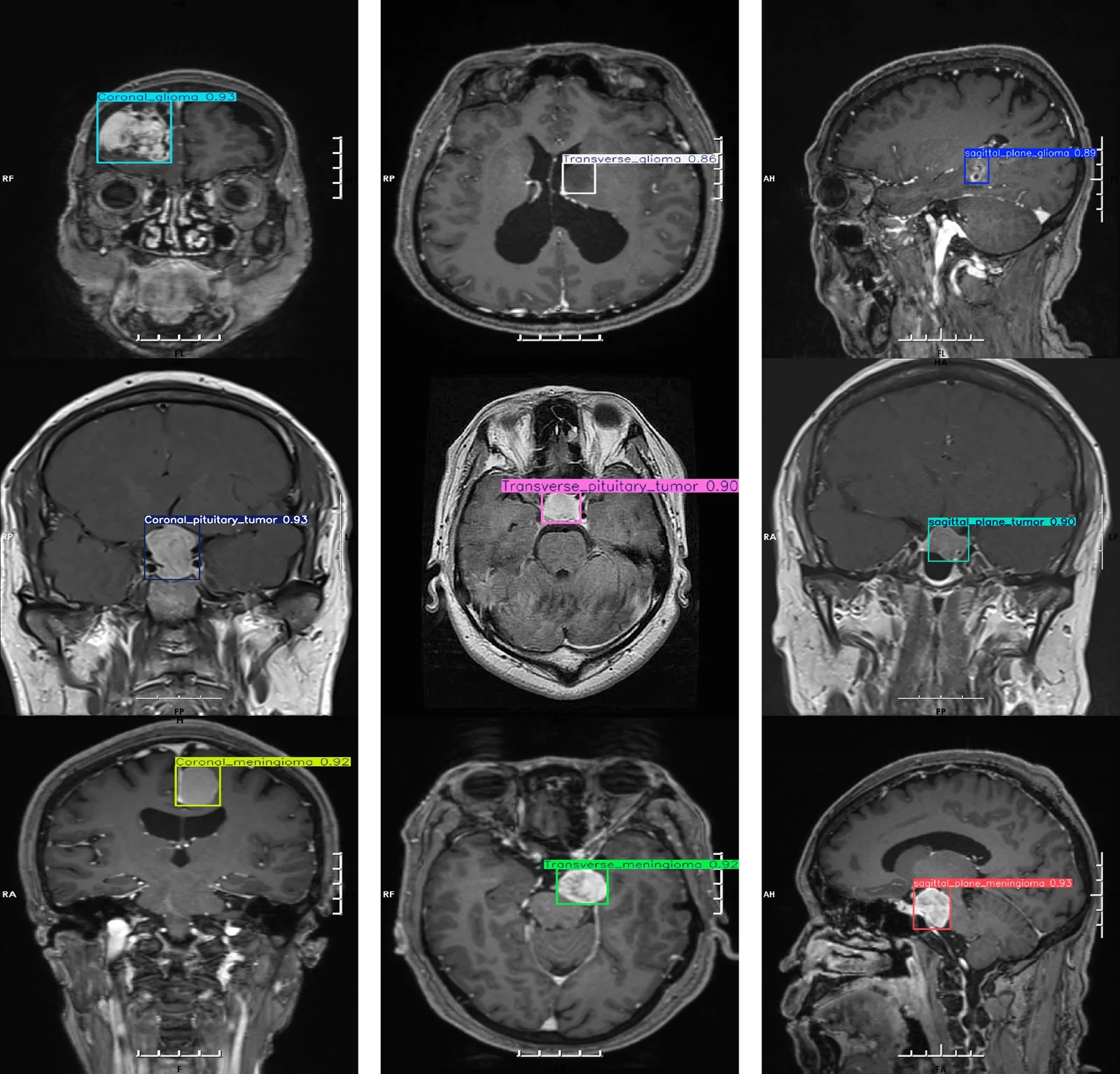
Detection results of brain tumors.

Overall, the FDANet model demonstrated excellent performance in the brain tumor detection task, with detection results illustrated in **Figure 6**. The clinical deputy chief physician has verified the authenticity of the detection results, confirming that they are consistent with the actual situation. The average precision and recall across all categories reached 0.960 and 0.952, respectively, indicating that the model possesses exceptionally high precision and recall rates. It can effectively localize tumor regions in medical images while keeping false positives (misdetections) and false negatives (missed detections) at extremely low levels. Furthermore, the overall mAP_50_ reached a high of 0.961, further confirming the model’s outstanding detection accuracy at an IoU threshold of 0.5. As a more stringent evaluation metric, the mAP_50–95_ also attained 0.874, demonstrating that the model not only performs well under lenient conditions but also maintains high localization and classification stability across varying overlap requirements, exhibiting strong robustness.

A detailed breakdown by tumor type and corresponding imaging plane provides deeper insight into the model’s performance characteristics:

1. Glioma Detection: The model performed best on glioma detection in the sagittal plane, achieving a perfect precision of 1.000 and a high mAP_50_ of 0.995. In comparison, precision on the coronal and transverse planes decreased slightly (0.896 and 0.918, respectively), although the mAP_50_ on the transverse plane remained high at 0.975. The recall for coronal gliomas (0.929) was slightly higher than its precision, suggesting that while the model misses very few gliomas in this plane, there may be a small number of false positives.
2. Pituitary Tumor Detection: For pituitary tumors, the model achieved perfect recall of 1.000 on both the sagittal and coronal planes, meaning all true tumors were successfully detected, with precision also near-perfect (>0.987). Although precision on the transverse plane was slightly lower (0.908), its recall remained remarkably high at 0.991. These results indicate the model’s exceptional capability in identifying pituitary tumors, with virtually no missed detections, particularly in the sagittal and coronal planes.
3. Meningioma Detection: Detection of meningiomas was also highly effective. On the transverse plane, the model achieved a precision of 0.979 and a recall of 0.948, with an mAP_50–95_ of 0.903—the highest comprehensive performance on the transverse plane among all tumor types. Precision on the sagittal and coronal planes remained above 0.976, demonstrating the model’s high confidence in meningioma detection.

#### 4.3.2 Ablation experiments

To thoroughly investigate the specific contributions of each proposed improvement module to the performance of the FDANet model, this paper designed and conducted a series of ablation experiments. All experiments were performed under identical dataset and training configurations, evaluating the model’s performance after the introduction of MANet (Mixed Aggregation Network), FasterCGLU (a lightweight module combining convolution and gated linear units), and WFU (Wavelet Feature Upgrade), respectively, with detailed results presented in **Table 4**.

**Table 4:** Impact of each improved module on overall model performance.

Model	Precision	Recall	mAP_50_	mAP_50-95_
Baseline (YOLOv11)	0.906	0.904	0.901	0.843
+MANet	0.916	0.923	0.914	0.853
+MANet+FasterCGLU	0.939	0.947	0.936	0.932
+WFU	0.929	0.925	0.921	0.883
+FasterCGLU	0.934	0.939	0.923	0.862
+MANet +FasterCGLU +WFU (This paper)	0.96	0.952	0.961	0.874

The baseline YOLOv11 model achieved a precision of 0.906 and a recall of 0.904 on the brain tumor MRI detection task, with mAP_50_ and mAP_50–95_ scores of 0.901 and 0.843, respectively, providing a reliable benchmark for subsequent improvements. After introducing the MANet module, all evaluation metrics showed steady improvement. Precision increased to 0.916, recall to 0.923, and mAP_50_ and mAP_50–95_ reached 0.914 and 0.853, respectively. This gain stemmed from MANet’s multi-path hybrid aggregation design: the depthwise separable convolution path is sensitive to textural details of small tumors, the standard convolution path provides stable semantic context, and the residual chain preserves shallow information through layer-wise refinement. The fusion of these three paths enables the model to adaptively capture critical spatial details of tumors, demonstrating enhanced discriminability for lesions with blurred boundaries. Further incorporating the FasterCGLU module on top of MANet (YOLOv11-MANet-FasterCGLU) led to continued improvements in Precision, Recall, and mAP_50_, which rose to 0.939, 0.947, and 0.936, respectively. Notably, the mAP_50–95_ of this two-module combination reached 0.932, substantially higher than the baseline and other intermediate configurations, indicating that the synergy between MANet and FasterCGLU is particularly effective under strict localization requirements. The gated linear unit in FasterCGLU enhances feature responses in tumor regions through a learnable gating mechanism while suppressing background noise (such as normal brain tissue or artifacts), which complements MANet’s detail extraction capabilities. Using the WFU module alone (YOLOv11-WFU) also demonstrated positive effects: precision reached 0.929, recall 0.925, mAP_50_ 0.921, and mAP_50-95_ 0.883. WFU adaptively adjusts contribution weights based on the importance of features at different levels through a learnable weighted fusion strategy, optimizing the integration of multi-scale information. The improvement was particularly notable at higher IoU thresholds (mAP_50-95_), indicating its positive impact on fine-grained localization. Applying the FasterCGLU module independently also yielded gains: precision of 0.934, recall of 0.939, mAP_50_ of 0.923, and mAP_50–95_ of 0.862. The gating mechanism’s selective enhancement of features effectively improved detection accuracy. However, limited by the feature extraction capability of a single module, its overall gain was slightly lower than when combined with MANet.

The complete FDANet, integrating all three modules, achieved the highest values among all tested configurations in the primary detection metrics of Precision, Recall, and mAP50. Precision reached 0.960, Recall 0.952, and mAP_50_ 0.961, representing the highest values across all tested configurations. However, its mAP_50–95_ was 0.874, which is lower than the MANet+FasterCGLU combination (0.932) and the WFU-alone configuration (0.883). This suggests that while the three modules jointly optimize detection confidence and coarse-grained localization (as reflected by mAP_50_), a trade-off exists in strict localization performance at high IoU thresholds. Nevertheless, the complete model maintains a mAP_50–95_ of 0.874, which still represents a 3.1 percentage point improvement over the baseline.

The complete FDANet achieves a higher mAP_50_(0.961) than the MANet+FasterCGLU configuration (0.936) but a lower mAP_50-95_ (0.874 vs. 0.932). This trade-off stems from the WFU module’s noise suppression. It filters out high-frequency background details via wavelet transform, improving coarse detection (mAP_50_) by reducing false positives, yet inevitably smooths some edge information critical for precise boundary alignment at high IoU thresholds. Consequently, the MANet+FasterCGLU configuration without WFU retains sharper edges and excels under strict localization requirements. Ablation results (**Table 4**) confirm that WFU alone also yields a higher mAP_50-95_ (0.883) than the complete model, indicating that this effect is inherent to WFU. Thus, FDANet’s lower mAP_50–95_ reflects a deliberate design choice prioritizing overall detection reliability over extreme localization precision.

To intuitively evaluate the model’s ability to focus on lesion regions, this study employed Grad-CAM to generate heatmaps for visual analysis. In the heatmaps, red indicates areas where the model’s attention is highly concentrated, while blue represents regions with lower attention.

**Figure 7:**
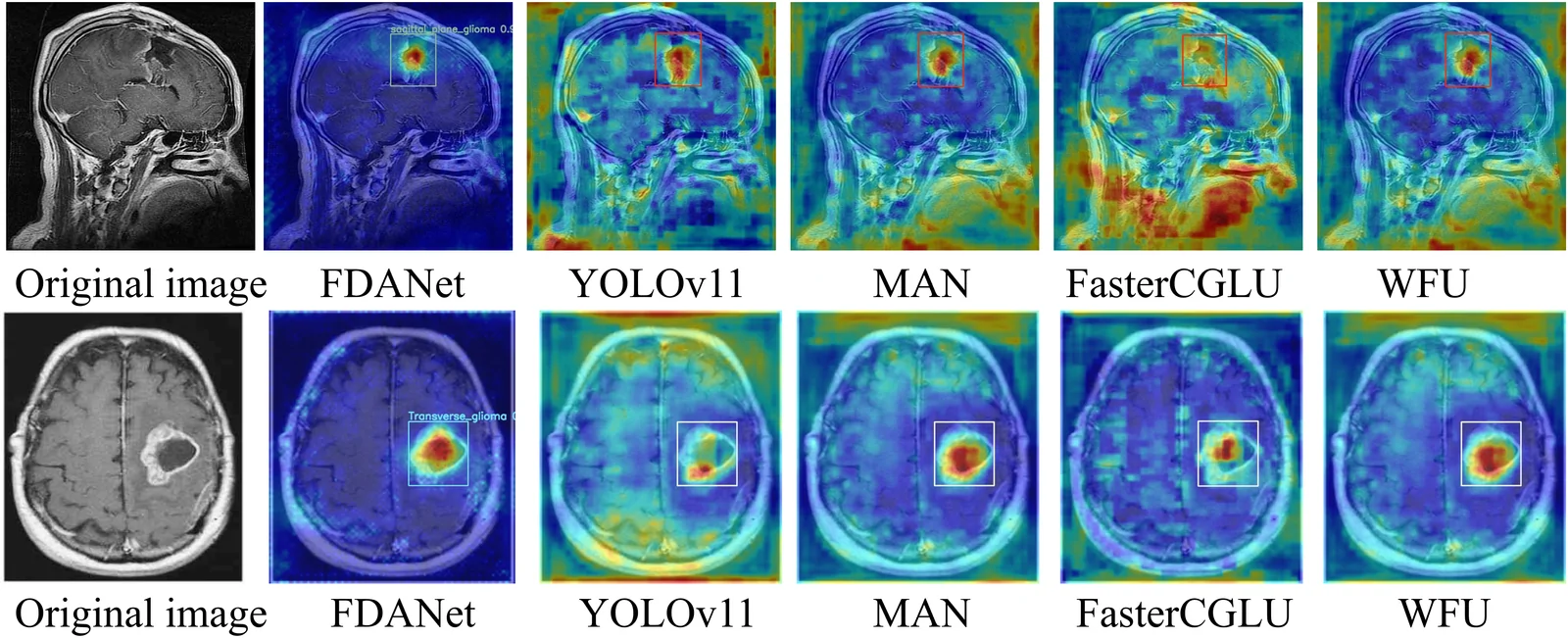
Grad-CAM heatmap visualization.

As shown in **Figure 7**, in the heatmap generated by the baseline YOLOv11, the red regions are relatively dispersed, extending beyond the tumor area into surrounding normal brain tissue. This indicates that the model is susceptible to interference from background noise. In contrast, the heatmap of FDANet shows red regions highly concentrated within the lesion itself, with clear boundaries that closely align with the actual tumor contour, while the background appears largely blue. The Grad-CAM visualizations were independently reviewed by an associate chief physician (the second author), who confirmed that the highlighted regions correspond to clinically relevant tumor boundaries and that FDANet’s attention maps show superior spatial alignment with lesion contours compared to the baseline.

This concentrated attention pattern can be attributed to the synergistic effect of multiple modules. MANet captures fine-grained spatial details, FasterCGLU enhances feature responses in lesion regions while suppressing noise through its gating mechanism, and WFU adaptively fuses multi-scale features. The combined action of these three modules enables the model to efficiently lock its attention onto critical regions, thereby significantly improving detection accuracy and overall localization stability (with mAP_50_ increasing from 0.901 to 0.961).

#### 4.3.3 Comparison with state-of-the-art

To comprehensively evaluate the performance of the proposed model, this paper conducts comparative experiments between FDANet and current mainstream object detection models on the brain tumor MRI dataset. The compared models include YOLOv5, YOLOv8, YOLOv9c, YOLOv10n, and the RT-DETR series (r18, r34, r50). All models were trained and tested under identical training configurations, using Precision, Recall, mAP_50_, and mAP_50–95_ as evaluation metrics, with the number of parameters (Params) and computational cost (GFLOPs) also recorded.

The experimental results are presented in **Table 5**. The proposed FDANet achieves optimal performance across all accuracy metrics. Precision reaches 0.960, Recall of 0.952, mAP_50_ of 0.961, and mAP_50–95_ of 0.874. Compared to the baseline YOLOv11, mAP_50_ improves by 6.0 percentage points; compared to the strong-performing YOLOv10n, mAP_50_ is 1.4 percentage points higher; and compared to RT-DETR-r34, mAP_50_ exceeds by 1.2 percentage points. In terms of model complexity, FDANet has 4.40M parameters and 9.3 GFLOPs, significantly lower than YOLOv9c (21.15M, 82.7 GFLOPs) and the RT-DETR series (19.9M~42.0M, 57~129.6 GFLOPs), while only slightly higher than lightweight YOLO models. After integrating the Swin Transformer architecture into YOLOv11, the model achieves an mAP50 of 0.907. In contrast, FDANet outperforms the YOLOv11-Swin Transformer model. This demonstrates an excellent balance between accuracy and efficiency.

**Table 5:** Experimental results of different model comparisons.

Model	Precision	Recall	mAP_50_	mAP_50-95_	Param	Gflops
YOLOv11	0.906	0.904	0.901	0.843	2,583,907	6.3
YOLOv9c	0.916	0.939	0.948	0.862	21,152,363	82.7
YOLOv5	0.911	0.929	0.932	0.856	2,183,419	5.8
YOLOv8	0.936	0.943	0.933	0.863	2,686,123	6.8
YOLOv10n	0.937	0.932	0.947	0.871	2,266,923	6.5
YOLOv11-Swin Transformer (26)	0.883	0.855	0.907	0.708	29,717,269	77.6
RT-DETR-r18	0.93	0.925	0.932	0.841	19,883,316	57
RT-DETR-r34	0.946	0.935	0.949	0.855	31,118,561	88.8
RT-DETR-r50	0.922	0.928	0.945	0.864	41,972,603	129.6
FDANet(This paper)	0.96	0.952	0.961	0.874	4,399,963	9.3

In summary, the proposed model achieves significant improvements in brain tumor detection accuracy while maintaining high efficiency, validating its potential for application in real-world clinical scenarios.

#### 4.3.4 Generalization testing

To further evaluate the model’s generalization capability in real-world clinical scenarios, this study conducted independent testing using brain tumor MRI images collected from our hospital between 2018 and 2021. The test set comprises imaging data from patients with gliomas, pituitary tumors, and meningiomas, encompassing variations in imaging equipment, scanning parameters, and individual patient differences, designed to assess the model’s adaptability and stability on unseen data.

**Figure 8:**
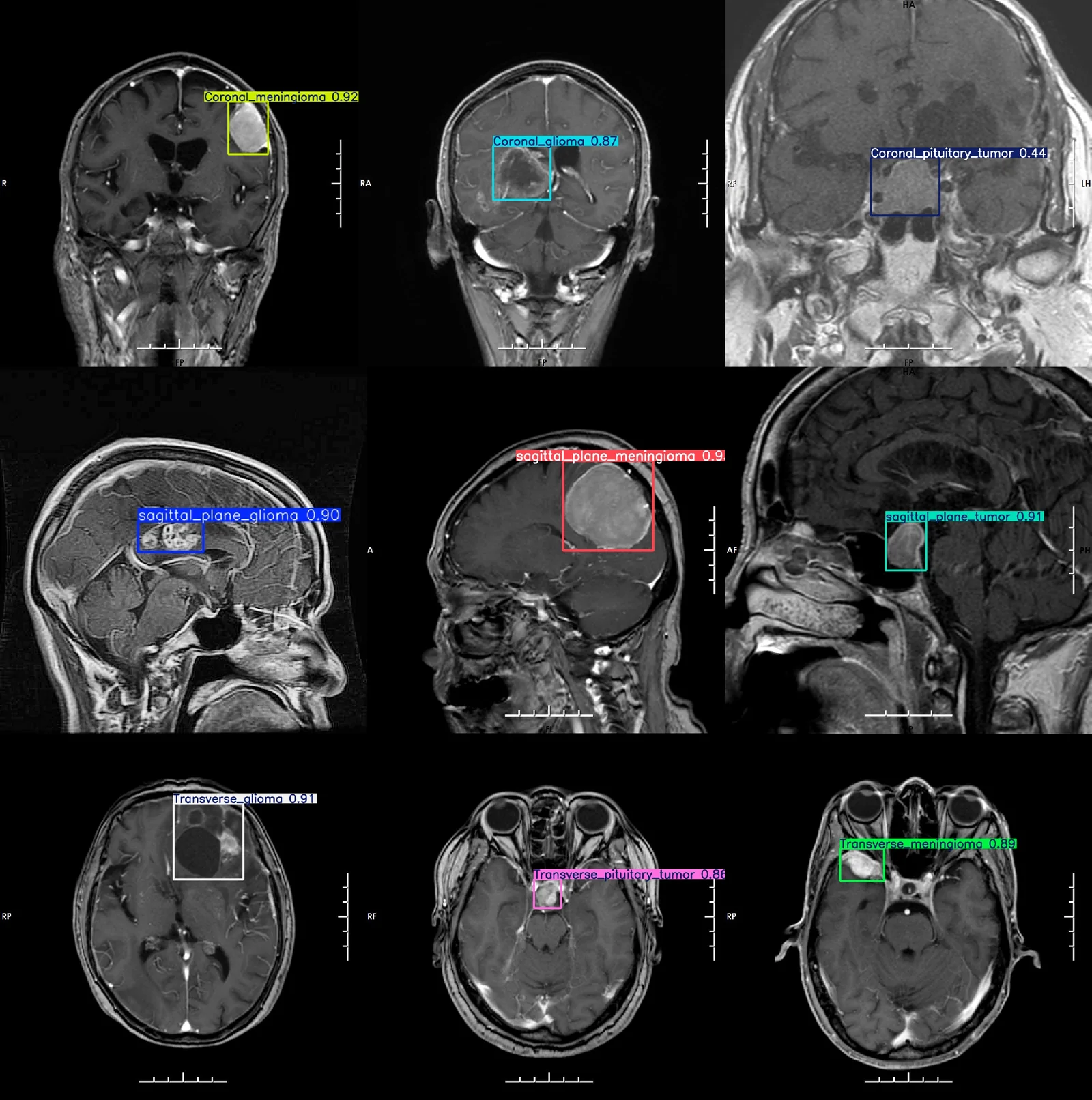
Detection results of FDANet on in-house brain tumor images.

**Figure 8** presents representative detection results. The clinical deputy chief physician has verified the authenticity of the detection results, confirming that they are consistent with the actual situation. As illustrated, the proposed FDANet model accurately identifies and localizes all three types of tumor lesions, with detection boxes closely aligning with the actual tumor boundaries. The model maintains stable detection performance even in cases involving irregular tumor morphology, blurred boundaries, or significant background interference. For multifocal lesions or small tumors, the model also demonstrates good sensitivity with low miss rates. Furthermore, the model achieves consistent detection performance across different imaging planes (transverse, coronal, and sagittal), indicating robustness to variations in image orientation.

Overall, the excellent performance of this model on our hospital’s clinical dataset validates its strong generalization capability and clinical applicability, laying a solid foundation for subsequent practical implementation.

#### 4.3.5 Five-fold cross-validation experiments of FDANet

To comprehensively evaluate the performance stability and statistical reliability of the proposed model, this paper calculated the mean, standard deviation, and 95% confidence intervals of each evaluation metric based on the 5-fold cross-validation results. As shown in **Table 6**, the mean values of Precision, Recall, mAP_50_, and mAP_50–95_ are 0.952, 0.951, 0.970, and 0.878, with corresponding standard deviations of 0.008, 0.001, 0.009, 0.004, respectively. These results sufficiently demonstrate the statistical reliability and reproducibility of the proposed model, indicating that the reported performance improvements rest on a solid statistical foundation.

**Table 6:** Five-fold cross-validation of FDANet.

K-folds	Precision	Recall	mAP_50_	mAP_50-95_
Fold 1	0.941	0.934	0.957	0.862
Fold 2	0.967	0.967	0.978	0.873
Fold 3	0.939	0.939	0.963	0.91
Fold 4	0.965	0.963	0.98	0.877
Fold 5	0.947	0.953	0.973	0.87
Average	0.952	0.951	0.97	0.878
standard deviation	0.008	0.001	0.009	0.004

## 5 Discussion

The proposed FDANet model exhibits exceptional detection capability on the brain tumor MRI dataset used in this study. For the vast majority of categories, both precision and recall exceed 0.90, with several metrics approaching or reaching unity. This outcome not only underscores the strong potential of the FDANet architecture in medical image analysis but also demonstrates its high suitability for multi-planar, multi-category brain tumor detection tasks. The observed performance variations across different imaging planes suggest that tumor morphology and its visual presentation influence detection results to some extent, thereby providing direction for subsequent data augmentation strategies or model fine-tuning.

Ablation experiments further reveal that FDANet achieves a precision of 0.960, a recall of 0.952, and an mAP_50_ of 0.961, substantially outperforming any two-module combination. This result indicates functional complementarity among the three core components. MANet focuses on extracting rich multi-scale details, FasterCGLU enhances tumor-relevant feature responses, and WFU optimizes cross-scale feature fusion. Ablation results confirm that the three modules are functionally complementary (**Table 4**). Their synergy yields higher performance than any two-module combination. It is worth emphasizing that the novelty of FDANet is not merely the sum of its modules. The key contribution lies in the synergistic design where MANet provides rich multi-scale features, FasterCGLU selectively enhances tumor-relevant information while suppressing noise, and WFU adaptively fuses cross-level representations. The ablation results (**Table 4**) confirm that the full combination yields substantially higher performance than any two-module pair, demonstrating true synergy rather than additive improvement.

Despite the progress achieved, certain limitations remain. First, the dataset used in this study covers only three tumor types (glioma, pituitary tumor, and meningioma), excluding other clinically relevant categories such as metastatic tumors and schwannomas. Future work will involve collecting additional tumor datasets to address this gap. Second, and more critically, the current FDANet framework operates on 2D MRI slices rather than volumetric 3D data, a design choice that warrants further discussion regarding its implications.

A potential limitation is the omission of volumetric context. Brain tumors, particularly gliomas, are spatially continuous structures that often infiltrate across multiple slices. By processing each slice independently, a 2D model inevitably loses inter-slice structural correlations. This shortcoming may lead to two specific issues: (i) small lesions appearing on only one or two consecutive slices may be missed due to the lack of contextual information from adjacent slices; and (ii) segmentation or bounding-box predictions may become inconsistent between neighboring slices, producing a “flickering” effect when the results are reconstructed into a 3D volume. For diffuse tumors lacking clear boundaries, the absence of volumetric context may also impair the model’s ability to differentiate tumor infiltration from normal tissue.

Rationale for adopting a 2D approach. Our choice is primarily dictated by the nature of the available datasets. Both the Alibaba Tianchi public dataset and our institutional collection consist of individual 2D slices with variable slice thicknesses and inconsistent inter-slice spacing, rendering direct 3D training infeasible. Moreover, a 2D model offers lower computational cost (9.3 GFLOPs and 4.40M parameters), which is advantageous for real-time clinical deployment and edge-device inference. Importantly, current radiological practice often involves scrolling through 2D slices sequentially; a high-performing 2D detector can still provide immediate clinical value by assisting slice-by-slice reading. The design ideas of this article (27) in terms of lightweight deployment and interpretability are highly relevant to this study, providing a reference example for future deployment of FDANet as a clinical real-time auxiliary diagnostic system. Meanwhile, future work will integrate clinical structured data with imaging unstructured data, for example the diagnosis of Alzheimer’s disease (28).

For future directions toward 3D analysis, we are actively exploring 3D extensions. We plan to collect or synthesize 3D-compatible MRI volumes with consistent slice spacing to enable such training.

## 6 Conclusion and future work

This paper systematically evaluates the performance of the proposed FDANet model for brain tumor MRI detection tasks through detection experiments, ablation studies, and comparative analyses, drawing the following conclusions:

1. In brain tumor detection experiments, the FDANet model demonstrated excellent detection performance. The model achieved an average precision of 0.960 and recall of 0.952 across all categories, with mAP_50_ reaching 0.961 and mAP_50–95_ attaining 0.874. For the three main tumor types—gliomas, pituitary tumors, and meningiomas—the model achieved near-perfect detection results across multiple imaging planes, validating its strong adaptability to different tumor types and imaging conditions.
2. Ablation experiment results indicate that progressively introducing MANet, FasterCGLU, and WFU modules based on YOLOv11 leads to continuous improvement in detection performance. The complete model achieved optimal results in Precision, Recall, mAP_50_, and mAP_50-95_, significantly outperforming the baseline and other module combinations. Heatmap visualization further revealed the synergistic effect of the three modules: while the baseline model’s attention was relatively dispersed, the complete model was able to focus attention highly on lesion regions while effectively suppressing background interference. These results fully validate the effectiveness of the multi-module collaborative design in enhancing brain tumor feature extraction and improving detection accuracy.
3. Comparative experiments further confirmed the superiority of the proposed model. Compared with current mainstream detection models (including YOLOv5, YOLOv8, YOLOv9c, YOLOv10n, and the RT-DETR series), FDANet achieved optimal performance across all four key metrics. Precision, Recall, mAP_50_, and mAP_50-95_. Specifically, mAP_50_ improved by 6.0 percentage points over the baseline YOLOv11 and exceeded the strong-performing YOLOv10n by 1.4 percentage points. Meanwhile, while maintaining high accuracy, the model has only 4.40M parameters and 9.3 GFLOPs, significantly lower than large models such as YOLOv9c and RT-DETR, demonstrating an excellent balance between accuracy and efficiency.

In summary, the FDANet model demonstrates effective lesion-focused attention and achieves improved accuracy and robustness in brain tumor MRI detection on a single-center 2D dataset while maintaining computational efficiency. These results suggest potential for clinical utility, but we emphasize that the current evidence is preliminary. Future work will involve prospective validation on multi-center, multi-modal, and volumetric datasets, as well as integration into clinical workflows, to establish the model’s real-world effectiveness.

## Data Availability

The original contributions presented in the study are included in the article/supplementary material. Further inquiries can be directed to the corresponding author.
